# MicroRNA-21 Identified as Predictor of Cancer Outcome: A Meta-Analysis

**DOI:** 10.1371/journal.pone.0103373

**Published:** 2014-08-06

**Authors:** Wenjie Zhu, Binghe Xu

**Affiliations:** Department of Medical Oncology, Cancer Hospital and Institute, Chinese Academy of Medical Sciences and Peking Union Medical College, Beijing, China; H. Lee Moffitt Cancer Center & Research Institute, United States of America

## Abstract

**Background:**

Growing evidence from recent studies has revealed the association of microRNA-21 (mir-21) with outcomes in multiple cancers, but inconsistent findings have been reported, which rationalized a summary and analysis of available data to investigate the prognostic role of mir-21.

**Materials and Methods:**

Eligible studies were identified through several search strategies and assessed for quality. Data was extracted from studies in terms of baseline characteristics and key statistics such as hazard ratio (HR), 95% confidence interval (CI) and *P* value, which were utilized to calculate pooled effect size.

**Results:**

25 studies were included in the meta-analysis to evaluate the prognostic role of mir-21 in malignant tumors. Elevated mir-21 level was demonstrated to moderately predict poor overall survival (OS) (HR = 1.903, 95% CI: 1.713–2.113, *P* = 0.000) and disease-free survival (DFS) (HR = 1.574, 95% CI: 1.139–2.175, *P* = 0.006) by the fixed and random effect model respectively. Importantly, subgroup analysis disclosed significant association between increased mir-21 level in cancerous tissue and worse survival status. Furthermore, over-expression of mir-21 was an independent prognostic factor for non-small cell lung cancer (NSCLC) and pancreatic cancer patients, with the pooled HR being 2.153 (95% CI: 1.693–2.739, *P* = 0.000) and 1.976 (95% CI: 1.639–2.384, *P* = 0.000).

**Conclusions:**

Over-expression of mir-21, especially in cancerous tissue, was effectively predictive of worse prognosis in various carcinomas. Non-invasive circulating mir-21, however, exhibited modest ability to discriminate outcomes. Major concerns about mir-21 assay standardization and selection of specimen need to be fully addressed before its practical implementation in management of cancer.

## Introduction

MicroRNAs (miRNAs) represent an evolutionarily conserved class of endogenous small non-coding RNA molecules that are approximately 18–25 nucleotides in length and post-transcriptionally modulate gene expression in a sequence-specific manner [1]–[3]. These tiny regulators mainly function via pairing with complementary sites within the 3′ untranslated region (UTR) of target messenger RNAs (mRNAs), leading to either translational suppression or degradation of mRNAs [4]–[6]. It is estimated that miRNAs may potentially regulate up to 30% of all human protein-coding genes [7]. Since the initial identification of miRNAs in 1993 [4], dramatic progress has been made in revealing their role in vital biological processes such as cell proliferation, differentiation, apoptosis and cell cycle control [8], [9]. Over the last decade, profiling studies have identified miRNAs that are aberrantly expressed in a variety of human cancers, and their association with cancer has prompted the functional classification of miRNAs into oncogenic (oncomirs) and tumor suppressor miRNAs. Oncomirs generally inhibit the expression of tumor suppressor genes and/or genes that are involved in cell apoptosis and differentiation [10], [11], among which mir-21 has been the most extensively explored one in human neoplasms of various origins.

Evidence about the pro-neoplastic role of mir-21 from fundamental and clinical studies is encouraging. When highly expressed, mir-21 was observed to promote cellular proliferation, survival, invasion and migration in multiple cancer cell lines [12]–[14]. Meanwhile, knock-down of mir-21 by anti-sense oligonucleotides caused notable decrease in cancer cell survival in vitro and tumor growth in vivo in a murine xenograft model, which was accompanied by abated expression of anti-apoptotic protein Bcl-2 and enhanced apoptosis [15]–[20]. Ever since the first report about its over-expression in glioblastoma in 2005 [16], mir-21 has been found up-regulated in a wide range of malignancies [21]–[24], which indicated its diagnostic role of distinguishing cancer patients from healthy individuals. More importantly, recent studies investigating its association with cancer outcomes have disclosed the prognostic value of mir-21, with its high expression predicting worse survival status in malignancies including breast cancer, lung cancer, pancreatic cancer, colorectal cancer, and so on [25]–[31]. However, consensus has not been reached as to the reliability of mir-21 as a prognostic biomarker in cancer because of some opposite results [32]–[35]. Considering the weakness of an individual study, it’s necessary to perform a meta-analysis to address the inconsistence of literature by systematically summarizing available findings.

In the current study, we carried out a meta-analysis to assess the prognostic significance of mir-21 in cancer. Since inconsistent evidence existed about the association of mir-21 with pancreatic and NSCLC survival, we performed cancer-specific subgroup analyses to clarify the correlation of mir-21 with these two malignancies. As for colorectal cancer however, the vast majority of available studies demonstrated that mir-21 over-expression could predict poor survival in colorectal cancer, which made it less necessary to re-prove previous finding in our study. In addition, implications for future research and feasibility of application in clinical practice were also explored.

## Materials and Methods

The meta-analysis was conducted following the guidelines of the Meta-analysis of Observational Studies in Epidemiology group (MOOSE) [36].

### Search strategy and study selection

MEDLINE, EMBASE and the Cochrane library electronic databases were systematically searched to identify relevant studies published from 1966 to 19 December 2013 using the following three sets of key words and their combination: “microRNA 21”, “cancer OR carcinoma OR tumor OR neoplasm OR malignancy” and “prognosis OR survival OR mortality OR death OR relapse OR recurrence OR metastasis OR outcome”. The titles and abstracts of the publications were carefully reviewed. In addition, a manual search was performed using references from relevant literature to further identify eligible studies.

Eligible studies enrolled participants diagnosed with a certain type of solid tumor, which measured the expression of miR-21 in cancerous tissues or circulatory system and investigated the association between miR-21 expression level and survival status. Articles were excluded if they were non-English articles, review articles, letters, economic analyses, or laboratory studies. Other exclusion criteria included studies analyzing a set of miRNAs altogether, studies dividing patients according to non-dichotomous miR-21 expression levels and studies lacking key information such as hazard ratio (HR), 95% confidence interval (CI) and *P* value. When multiple publications about a study were identified, only those representing the latest reference and reporting the outcomes were included.

### Quality assessment

Following a critical review checklist of the Dutch Cochrane Centre proposed by MOOSE, we systematically assessed the quality of all the studies included [36]. Major items to be evaluated are as follows: (i) clear description of study population and origin of country, (ii) clear description of disease type, (iii) clear description of study design, (iv) clear definition of cancer outcomes, (v) clear explanation of measurement of miR-21, (vi) clear definition of cut-off value of miR-21 level and (vii) sufficient duration of follow-up. If a study failed to specify information concerning any aspect stated above, it would be excluded so as not to compromise the quality of the meta-analysis.

### Data extraction and conversion

Data was extracted from all eligible studies in duplicate by two independent reviewers. Disagreement was resolved by consulting with a third reviewer. Data was collected with regard to the following aspects: (i) publication details: the last name of first author, year of publication and study design; (ii) baseline characteristics of study population: country, sample size, site and staging of cancer; (iii) miR-21 assay specimen, method and cut-off value of mir-21 level and (iv) HR of increased miR-21 for overall survival (OS), relapse-free survival (RFS) or disease-free survival (DFS), as well as their 95% CI and *P* value. In most cases we directly derived HR and 95% CI from the original article, with an HR of >1 being associated with elevated risk of mortality or recurrence. If HR and 95% CI were absent, the total number of observed deaths or recurrences and the sample size in each group were extracted to calculate HR as previously described [37]. If only Kaplan–Meier curves were available, data was extracted from the survival plots and an estimated HR was then calculated as previously described [37].

### Statistical analysis

Heterogeneity among included studies was evaluated using Cochran’s Q test and Higgins I-squared statistic. A random effect model (Der Simonian and Laird method) was adopted as the pooling method if substantial heterogeneity was observed (*P*<0.05), while the fixed effect model was applied in the absence of between-study heterogeneity (*P*>0.05). Publication bias was assessed using the funnel plot with the Egger’s bias indicator test [38]. Sensitivity analysis (influence analysis) was performed to test how robust the pooled effect size was to the removal of individual studies. An individual study was suspected to have excessive influence if the point estimate was outside the 95% CI of the combined effect size after it was removed from the analysis. Subgroup analysis was conducted based on the type of specimen collected and the site of cancer. All of the *P* values were two-sided, with *P*<0.05 considered statistically significant. All analyses were performed using STATA version12.0 (Stata, College Station, TX, USA).

## Results

### Literature screening and study characteristics

**Figure 1:**
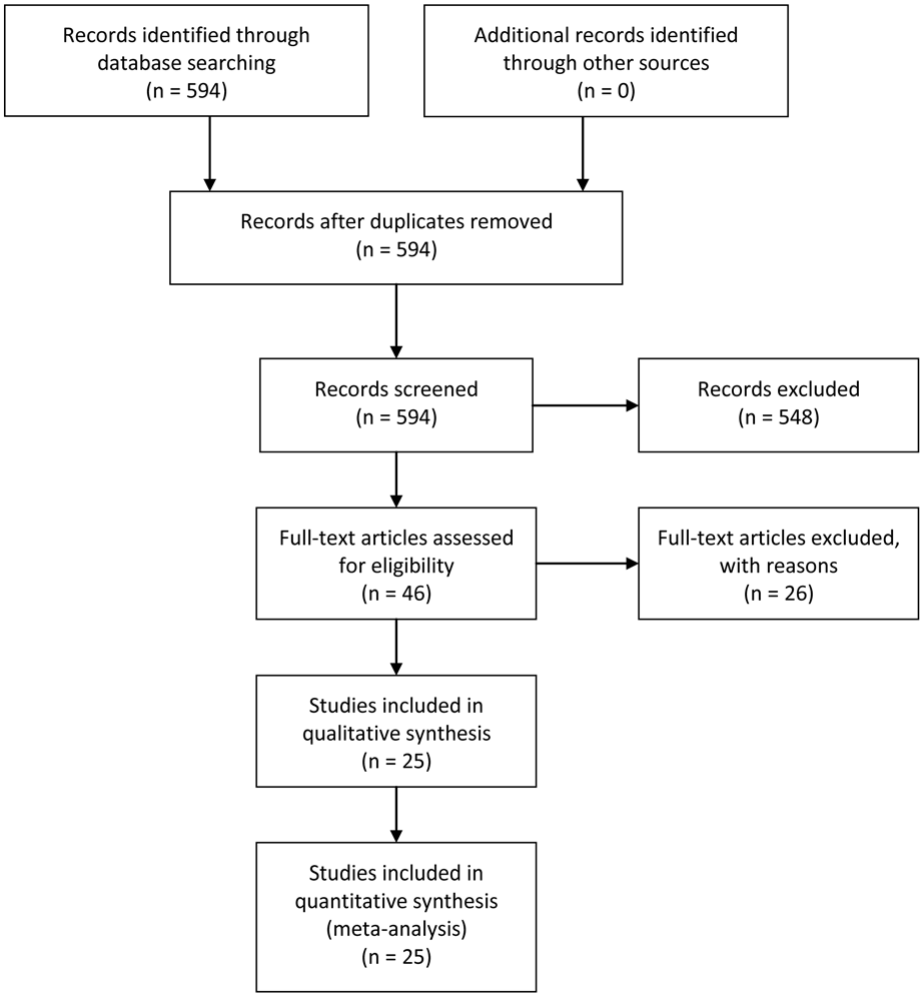
Flow chart of literature review and study selection process.

Figure 1 presented the process of literature screening and study selection. After preliminary on-line search, 594 original articles concerning mir-21 and cancer prognosis were retrieved out of EMBASE, MEDLINE and the Cochrane library databases. 548 papers were excluded from the present study after manual screening of titles, abstracts and key words because they were review articles, letters, laboratory studies, non-English contributions or irrelevant to the current analysis. Full texts of the remaining 46 articles were carefully reviewed and assessed, and 22 articles were further removed due to non-dichotomous classification of mir-21 expression levels or lack of key statistics such as HR, 95% CI and *P* value. Of the 29 candidate studies from 24 published papers, one study evaluated a series of miRNAs as a whole [39], 1 article failed to provide definite information about cancer staging [40], and 2 studies were considered ineligible due to compromised generalizability since the data was derived from cancer patients restricted to a certain stage [41], [42]. Finally, 25 studies in all were included in the meta-analysis to evaluate the prognostic role of mir-21 in malignant tumors.

The main characteristics and basic information of eligible studies were summarized in Table S1. The studies enrolled 3,038 patients from the United States, the United Kingdom, Canada, Italy, Norway, Greece, Denmark, China, Taiwan and Japan. All the studies were retrospective, which dealt with a wide range of carcinomas including breast cancer, colorectal cancer, lung cancer, pancreatic cancer, melanoma, glioma, gastric cancer, oral cancer, hepatocellular carcinoma, renal cell carcinoma and prostate cancer. The majority of the studies examined the expression of mir-21 in cancerous tissue, yet 6 studies targeted blood serum as a source of interest. Notably, in one study Ota and his colleagues sought to quantify the level of mir-21 in bone marrow obtained from breast cancer patients. In situ hybridization (ISH) was applied in two studies although quantitative real-time PCR (qRT-PCR) remained as the predominant way of mir-21 detection. 15 studies adopted the median fold change as the cut-off value of mir-21 expression, with 2-fold, 5-fold and mean fold change used in the other studies. 7 of the 25 studies explored the association of mir-21 with disease-free survival (DFS), relapse-free survival (RFS) or time to progression (TTP), and 18 focused on its relation with overall survival (OS) of cancer patients.

### Mir-21 and overall survival

**Figure 2:**
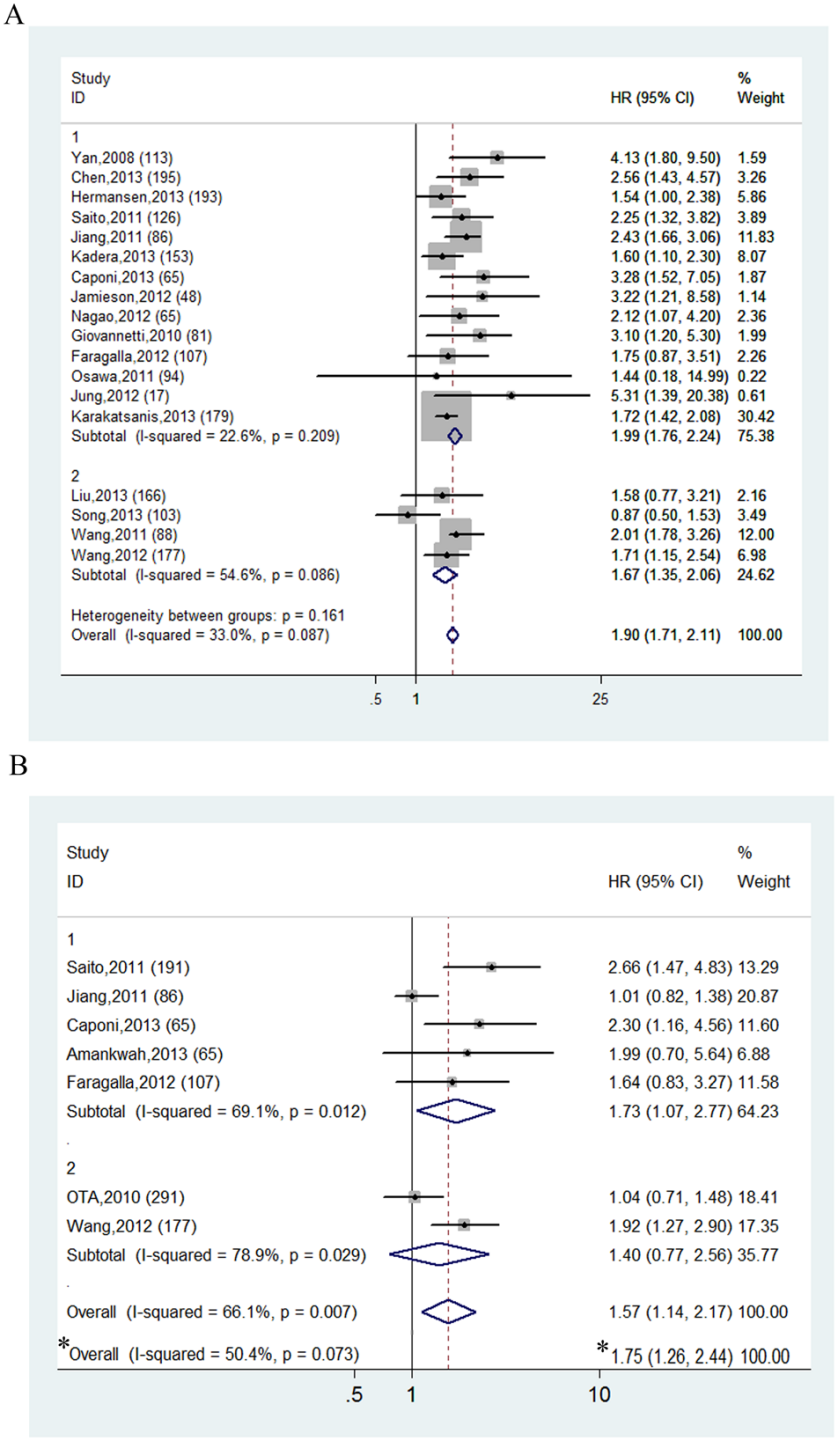
Forest plots of the analyses about mir-21 and overall survival (OS) (A) and disease-free survival (DFS) (B). Fixed (A) and random (B) effect model was used as the pooling method respectively. Studies are stratified based on the type of specimen: 1 for cancerous tissue and 2 for circulating mir-21. * Analysis about mir-21 and DFS after the omission of the study by Jiang et al.

Figure 2A displayed the forest plot of the analysis about mir-21 and OS. 18 studies in all were subjected to analysis. The fixed effect model was utilized to calculate the pooled effect size due to absence of heterogeneity among the studies (I^2^ = 33.0%, *P* = 0.087). Mir-21 high expression was demonstrated to moderately predict poor OS regardless of the site of cancer (HR = 1.903, 95% CI: 1.713–2.113, *P* = 0.000). Afterwards we carried out subgroup analyses according to the types of sample collected for mir-21 assay, namely cancerous tissue and blood serum. For the 14 studies using frozen or fresh tissues, by the fixed effect model the pooled HR for OS was 1.986 (95% CI: 1.760–2.241, *P* = 0.000), suggesting that over-expression of mir-21 in cancer tissues was predictive of worse outcome. As for the 4 studies targeting serum mir-21, it turned out that elevated mir-21 level in serum was associated with undesirable prognosis, with the combined HR for OS being 1.669 (95% CI: 1.351–2.062, *P* = 0.000).

### Mir-21 and disease-free survival

Figure 2B showed the analysis results of 7 studies about DFS. Evident heterogeneity was detected among the seven studies (I^2^ = 66.1%, *P* = 0.007), which rationalized further exploration to disclose factors contributing to this heterogeneity. The combined HR was calculated to be 1.574 (95% CI: 1.139–2.175, *P* = 0.006) by the random model, providing hints that increased expression of mir-21 was correlated with reduced DFS of cancer patients.

### Subgroup and sensitivity analysis of DFS studies

In order to specify the source of heterogeneity the DFS studies were stratified based on the types of specimen, yet prominent heterogeneity was again identified within both groups (2 studies in circulating mir-21 group I^2^ = 78.9%, *P* = 0.029; 5 studies in tissue mir-21 group I^2^ = 69.1%, *P* = 0.012)(Fig. 2B). However, substantial heterogeneity could also be attributed to the wide-ranging origins of study cohorts, so the studies were then entered into subgroup analysis by countries of the study population (Asian and non-Asian countries). To a huge extent heterogeneity was dissolved within three non-Asian studies (I^2^ = 0.0%, *P* = 0.791) rather than the four Asian studies (I^2^ = 78.5%, *P* = 0.003), and elevated mir-21 manifested itself as more indicative of shortened DFS in non-Asian cancer patients (HR = 1.951, 95% CI: 1.257–3.027, *P* = 0.003) than Asian cohorts (HR = 1.445, 95% CI: 0.952–2.193, *P* = 0.084).

Figure S1 exhibited the results of sensitivity analysis, through which it was uncovered that the study by Jiang et al [43] had excessive influence over the pooled HR for DFS of all cancers. After exclusion of the study by Jiang et al, heterogeneity among DFS studies was significantly diminished (I^2^ = 50.4%, *P* = 0.073), and no eminent difference was observed between the newly derived pooled HR (1.753, 95% CI: 1.258–2.442, *P* = 0.001) and the original one (1.574, 95% CI: 1.139–2.175, *P* = 0.006), which implied the combined result was considerably robust (Fig. 2B).

### Cancer-specific analysis

**Figure 3:**
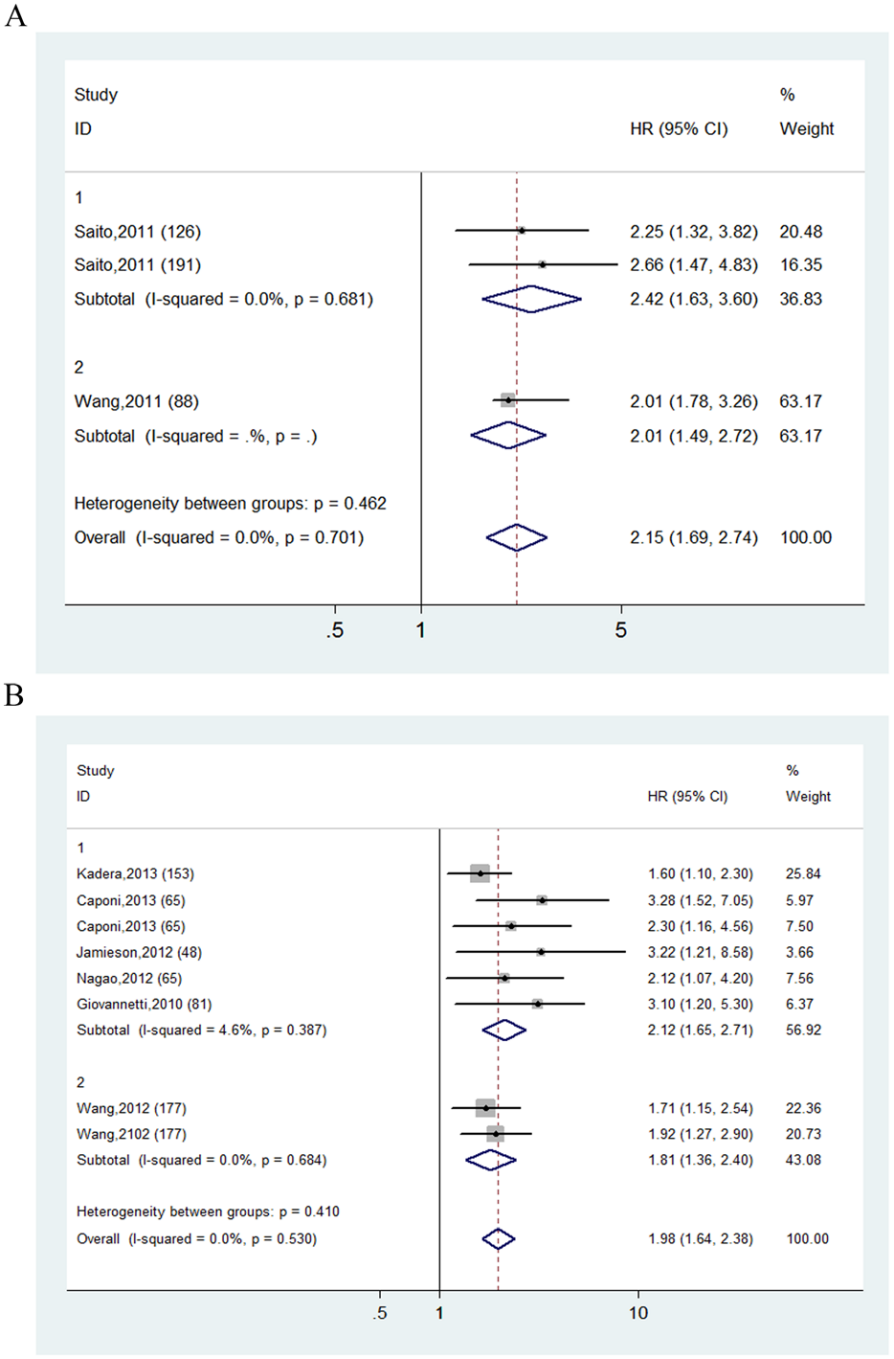
Forest plots derived from the analyses of non-small cell lung cancer (NSCLC) (A) and pancreatic cancer (B) studies. Fixed effect model was used as the pooling method. Studies are stratified based on the type of specimen: 1 for cancerous tissue and 2 for circulating mir-21.

Subsequently we set out to throw light upon the prognostic role of mir-21 in certain cancers. Figure 3A represented the forest plot of the analysis of non-small cell lung cancer (NSCLC) studies. Three studies enrolling 405 patients from USA, China, Japan and Norway went through analysis. Inter-study heterogeneity was absent (I^2^ = 0.0%, *P* = 0.701). Obviously over-expression of mir-21 was an independent prognostic factor for NSCLC patients, with the pooled HR being 2.153 by fixed effect model (95% CI: 1.693–2.739, *P* = 0.000). When it came to pancreatic cancer, 8 studies involving 831 participants from USA, the UK, Italy, China and Japan were put to analysis, which displayed great homogeneity (I^2^ = 0.0%, *P* = 0.530). Fixed effect model was applied and the pooled HR was 1.976 (95% CI: 1.639–2.384, *P* = 0.000), revealing significant association between increased mir-21 level and worse pancreatic cancer survival status (Fig. 3B). Additionally, the studies on NSCLC and pancreatic cancer were further stratified by sample type, and it was discovered that both tumor and circulating mir-21 over-expression remained predictive of undesirable cancer outcome.

### Publication bias

**Figure 4:**
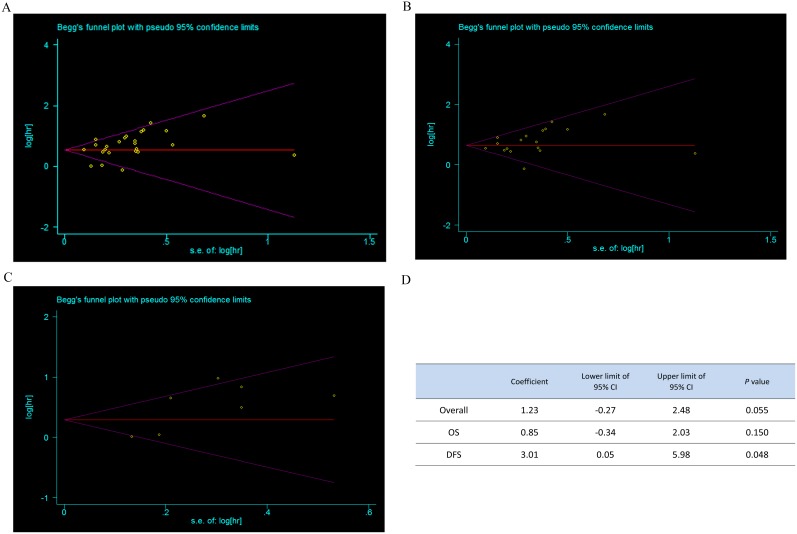
Publication bias of the included studies. Funnel plots provided graphic estimate of bias for overall studies (A), OS studies (B) and DFS studies (C) respectively. Main statistics of Egger’s test are summarized (D).

Finally, publication bias of the included studies was assessed by funnel plot and Egger’s test, which was summarized in Figure 4. Figure 4D exposed the results of Egger’s test, which was used to provide statistical evidence of funnel plot symmetry. Publication bias was not detected in the overall analysis of 25 enrolled studies (*P* = 0.055). For the eighteen OS studies Egger’s test revealed no evidence of publication bias (*P* = 0.150) while with seven DFS studies publication bias existed (*P* = 0.048). Similar results were demonstrated in the funnel plot (Fig. 4A–C).

## Discussion

In the current meta-analysis which pooled global high-quality studies concerning mir-21 and cancer prognosis, it was demonstrated that over-expression of mir-21, especially in cancerous tissues, was effectively predictive of poor survival in a variety of cancers, in terms of both OS and DFS. For NSCLC and pancreatic cancer, significant association was verified between elevated mir-21 level and worse long-term survival.

The studies setting OS as primary endpoint were largely homogeneous, which made it relatively safe to conclude that high level of tissue or circulating mir-21 was predictive of reduced OS. Nevertheless caution should be taken when interpreting the analysis results of the DFS studies, which exhibited prominent heterogeneity. In the subgroup analysis by types of sample, mir-21 level in cancerous tissues (HR = 1.725, 95% CI: 1.073–2.775, *P* = 0.024) rather than blood serum (HR = 1.403, 95% CI: 0.769–2.558, *P* = 0.269) was associated with decreased DFS in multiple cancers. To sum it up, promoted mir-21 level in cancer tissues was related to greater risk of relapse and mortality while the predictive force of circulating mir-21 only applied to OS given the analysis of available studies.

Since the initial recognition of the association of mir-21 with cancer in 2005 [16], mir-21 has stood out as the most extensively explored miRNAs. Recent studies involving cancer cell lines and xenograft models have implicated the oncogenic role of mir-21. Accumulating evidence has supported mir-21 as a potential diagnostic and prognostic biomarker in various carcinomas. In our meta-analysis study, it was preliminarily concluded that mir-21 level facilitated the prediction of long-term survival in cancers including NSCLC and pancreatic cancer, which further validated the prognostic value of this oncomir. As a matter of fact, the noteworthy association between aberrant mir-21 expression and poor survival could be best illuminated by its indispensable role in carcinogenesis and metastatic cascade. Latest findings have shed light upon the underlying oncogenic mechanisms of mir-21, which exerted profound influence over the basic hallmarks of cancer, including cell proliferation, apoptosis, differentiation, invasion and migration [8]–[11]. The mir-21 gene is located on chromosome 17q23.2 within the common fragile site FRA17B, which is frequently observed to be amplified in numerous malignancies [21]. Of great importance, the oncogenic effect of mir-21 could be primarily explained by its transcriptional targets and downstream signal pathways. So far, validated targets of mir-21 included programmed cell death 4 gene (PDCD4), tropomyosin 1 (TPM1), phosphatase and tensin homolog (PTEN), chromosome condensation protein G (NCAPG), reticulon 4 isoform A (RTN4) and other cancer-related genes [12]–[15], [17], [44]–[46]. Altogether, the vital cellular pathways regulating cell proliferation, apoptosis and cell cycle such as Ras [47], p53 [48], PI3K-Akt-mTOR pathway [49], as well as target genes compose an intricate network of mir-21 modulation.

As mounting evidence from retrospective studies indicated mir-21 as a promising tumor marker, a series of quantitative analyses were carried out based on published studies to help determine its diagnostic and prognostic value. A meta-analysis by Xia et al demonstrated that in colorectal cancer high-level mir-21 was moderately predictive of unfavorable overall survival (HR = 1.76, 95% CI: 1.34–2.32, *P* = 0.000) [50]. On the basis of 17 studies, Fu et al reported in a systematic review and meta-analysis that mir-21 over-expression predicted worse overall and relapse-free survival in several cancers including head and neck squamous cell carcinoma (HNSCC) and digestive system cancers, with the pooled HR being 1.69 (95% CI: 1.33–2.16, *P*<0.001) for OS and 1.48 (95% CI: 1.03–2.11, *P*<0.033) for DFS [51]. Similar results were obtained by Wang et al in their meta-analysis about the prognostic significance of circulating mir-21 in human cancer, which included six studies and estimated the combined HR to be 2.37 (95% CI: 1.83–3.06, *P* = 0.000) [52]. The data derived from studies stated above however, should be translated with caution due to dramatic across-study heterogeneity and small sample capacity, which justified further analysis to validate the prognostic role of mir-21. Compared to previous meta-analyses, the current one involved as many as 25 high-quality studies including numeral newly published ones, most of which focused on pancreatic cancer. As a matter of fact, opinions are divided as to the association of mir-21 expression with pancreatic cancer outcome and in latest years extensive translational research has cast light upon this dispute. In the present work, we conducted subgroup analysis of pancreatic cancer studies, which added convincing evidence that mir-21 over-expression predicted reduced survival regardless of its sample type. Notably, we set and implemented exacting inclusion and exclusion criteria to ensure the quality of involved studies and thus reliability of the pooled results. For instance, if a study dealt with s series of miRNAs and failed to evaluate each miRNA for its correlation with survival independently, it wouldn’t be selected for being less representative and relevant to our research. Distinct from earlier meta-analyses, the present work paid substantial attention to the details of study design and data reporting in quality assessment. A study would be presumed to exhibit compromised generalizability and then rejected if it failed to specify the cancer stage of its participants or it merely involved patients of one certain stage. Moreover, in order to guarantee the homogeneity of enrolled studies and consequently the reliability of pooled result two studies [42], [53] that measured miR-21 expression continuously were excluded, but both studies reported that high levels of miR-21 was related with poor survival in cancer, which was consistent with our finding in the current meta-analysis. Of importance, the included OS and DFS studies were both put to stratified analysis according to the sources of mir-21. To our knowledge, our study was unique in adopting that subgroup analysis and supplied initial quantitative evidence about the prognostic value of circulating and tissue mir-21 respectively. Besides, in the presence of heterogeneity among DFS studies, we performed further sensitivity and subgroup analysis, which was obviously absent from earlier relevant meta-analyses, to track down the origin of heterogeneity. By that means we fulfilled a comprehensive exploration and arrived at objective and unbiased conclusions.

Nonetheless the present meta-analysis study does have several limitations. To start with, substantial heterogeneity existed among the DFS studies, which was mainly caused by a relative paucity of studies evaluating DFS as an outcome. The divergence of incorporated studies was also probably ascribed to the difference in the demographics of participants, site of cancer, duration of follow-up, type of specimen, method of assay and cut-off value of mir-21 level. In the subgroup analysis of DFS studies, heterogeneity was eliminated in the non-Asian studies (I^2^ = 0.0%), and it was discovered that increased mir-21 expression was correlated with shortened DFS in non-Asian cancer patients. In order to minimize the confounding influence of heterogeneity, random effect model was used in the pooling of effect size. Moreover, in the final conclusion of our analysis we failed to specify the definition of mir-21 over-expression due to the inconsistency in the cut-off values of mir-21 level among the included studies. Furthermore, the analysis results of the NSCLC studies should be interpreted with caution because of a relatively small sample size (405 participants). Additionally, in the analysis of DFS studies the research by OTA et al, which fell into the circulating mir-21 subgroup, measured mir-21 level in bone marrow and the derived result might not appropriately apply to circulating mir-21. Last but not least, publication bias was detected in the DFS studies, indicating that studies with positive findings were more likely to be reported and published which might introduce over-estimate of the pooled HR.

Together with previous findings, our study has provided convincing evidence supporting mir-21 as a promising prognostic biomarker for cancer. Yet several considerations have to be delivered over its clinical application. First, priority must be given to the standardization of mir-21 assay procedure, including the methods of assay (qRT-PCR, ISH, IHC etc.), selection of internal reference RNA and most importantly, the cut-off value of mir-21 expression level. To a large extent, the divergence of contemporary findings about the prognostic value of mir-21 could be accredited to methodological inconsistency. The approach to setting cut-off value of mir-21 expression varied among different studies, which is obviously the principal concern needed to be resolved prior to its clinical application. Determination of global patterns of mir-21 expression will significantly prompt achievement of final consensus. In addition, in our study circulating mir-21 only displayed modest ability to discriminate outcomes, though it has been actively proposed as a non-invasive indicator of prognosis for numerous malignancies in latest years. Dispute existed in abundance concerning the authentic efficacy of cell-free mir-21 in differentiating outcomes. According to Chen et al [54], extracellular mir-21 probably originated from normal and/or tumor-lysed cells in the body fluids, which definitely created interference to the results obtained. Further studies are necessary to address that discrepancy and clarify the prognostic value of circulating mir-21.

In summary, our study has demonstrated that over-expression of mir-21 was effectively predictive of worse prognosis in various carcinomas. Increased mir-21 level in cancerous tissues was associated with reduced OS and DFS while elevated circulating mir-21 was only indicative of poor OS. In future more clinical studies are warranted to confirm the prognostic role of mir-21 before its practical implementation in management of cancer.

## Supporting Information

Figure S1 **Sensitivity analyses of studies concerning mir-21 and DFS.**
(TIF)

Table S1 **Summary of the characteristics of enrolled studies.**
(DOCX)

Checklist S1 **PRISMA 2009 Checklist.**
(DOC)
