# Supplementary figures and images for: MicroRNA-21 Identified as Predictor of Cancer Outcome: A Meta-Analysis
Source: PLoS One. 2014 Aug 6;9(8):e103373. doi: 10.1371/journal.pone.0103373 (PMC4123876; doi:10.1371/journal.pone.0103373)

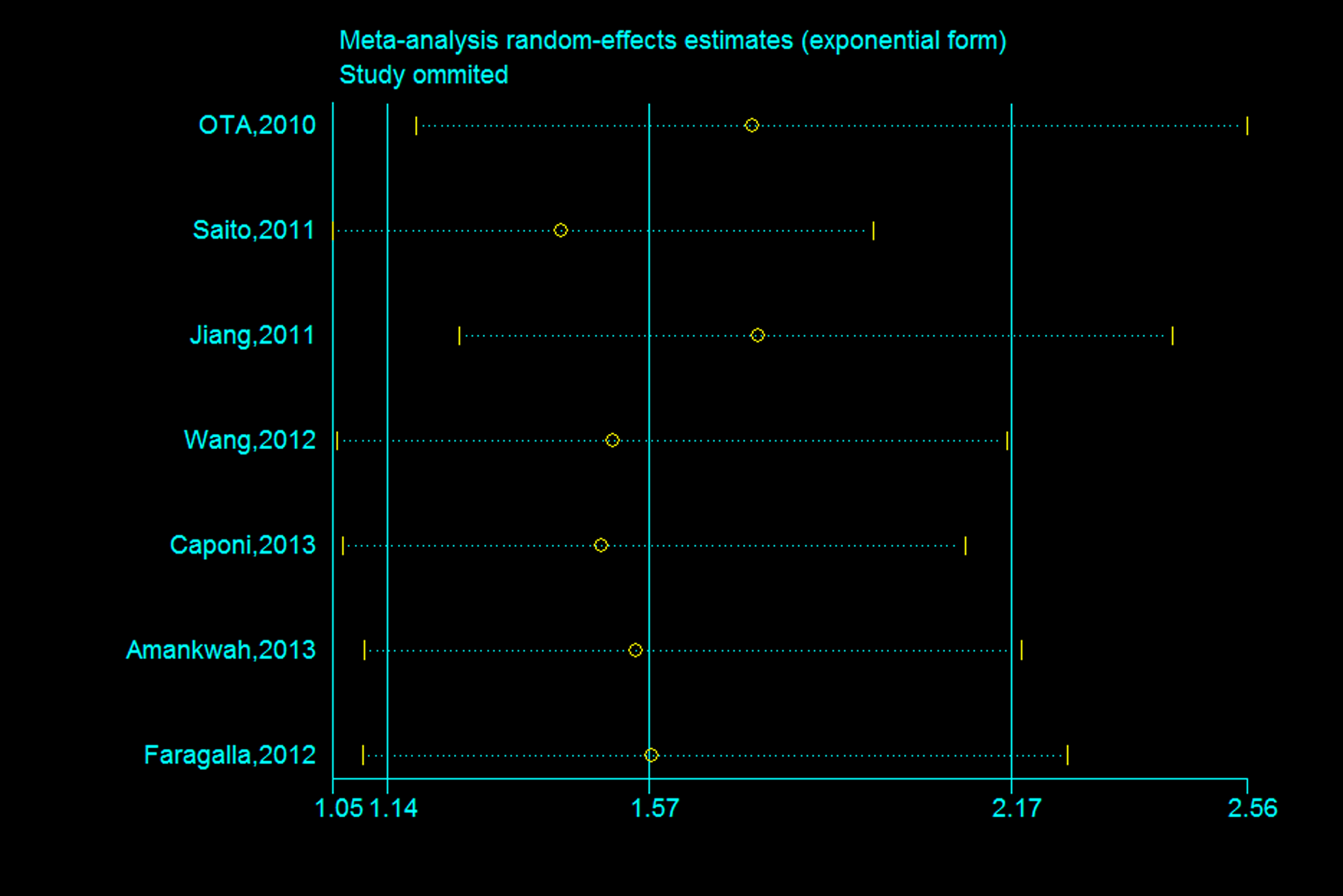

Supplement: Figure S1 — Sensitivity analyses of studies concerning mir-21 and DFS. (TIF) [file pone.0103373.s001.tif]
