# Supplementary material for: MicroRNA-21 Identified as Predictor of Cancer Outcome: A Meta-Analysis
Source: PLoS One. 2014 Aug 6;9(8):e103373. doi: 10.1371/journal.pone.0103373 (PMC4123876; doi:10.1371/journal.pone.0103373)
Supplement: Table S1 — Summary of the characteristics of enrolled studies. (DOCX) [file pone.0103373.s002.docx]

Table S1 Summary of the characteristics of enrolled studies

| **author** | **year** | **country** | **study design^a^** | **cancer** | ***N*** | **stage** | **Specimen** | **assay** | **cut-off value** | **endpoint** | **HR** | **lower limit** | **upper limit** | ***P* value** | **median duration of follow-up (months)** |
| --- | --- | --- | --- | --- | --- | --- | --- | --- | --- | --- | --- | --- | --- | --- | --- |
| Yan | 2008 | China | R | Breast cancer | 113 | I-III | Tissue | qRT-PCR | mean | OS | 4.133 | 1.799 | 9.499 | 0.001 | 66.2 |
| Chen | 2013 | Taiwan | R | Colorectal cancer | 195 | I-IV | Tissue | qRT-PCR | mean | OS | 2.56 | 1.433 | 4.574 | 0.002 | 48-96 |
| Liu | 2013 | China | R | Colorectal cancer | 166 | I-IV | Serum | qRT-PCR | 2-fold | OS | 1.58 | 0.77 | 3.21 | 0.126 | 36.4 |
| Song | 2013 | China | R | Gastric cancer | 103 | I-IV | Serum | qRT-PCR | median | OS | 0.873 | 0.498 | 1.53 | 0.873 | 35.9 |
| Hermansen | 2013 | Denmark | R | Glioma | 193 | I-IV | Tissue | ISH | 2-fold | OS | 1.545 | 1.002 | 2.381 | 0.049 | 9-69 |
| Wang | 2011 | China | R | NSCLC | 88 | I-III | Serum | qRT-PCR | 5-fold | OS | 2.01 | 1.78 | 3.26 | 0.015 | 52.16 |
| Saito | 2011 | Maryland  Norway | R | NSCLC | 126 | I-III | Tissue | qRT-PCR | median | OS | 2.25 | 1.32 | 3.82 | 0.003 | 80 |
| Jiang | 2011 | China | R | Melanoma | 86 | I-IV | Tissue | qRT-PCR | median | OS | 2.435 | 1.662 | 3.057 | 0.028 | 60 |
| Kadera | 2013 | USA | R | Pancreatic cancer | 153 | I-IV | Tissue | ISH | median | OS | 1.6 | 1.1 | 2.3 | 0.02 | 84 |
| Wang | 2012 | China | R | Pancreatic cancer | 177 | III-IV | Serum | qRT-PCR | median | OS | 1.705 | 1.147 | 2.535 | 0.008 | 20 |
| Caponi | 2013 | Italy UK | R | Pancreatic cancer | 65 | I-III | Tissue | qRT-PCR | median | OS | 3.28 | 1.52 | 7.05 | 0.02 | 28.4 |
| Jamieson | 2012 | UK | R | Pancreatic cancer | 48 | I-III | Tissue | qRT-PCR | median | OS | 3.22 | 1.21 | 8.58 | 0.019 | 23.9 |
| Nagao | 2012 | Japan | R | Pancreatic cancer | 65 | I-III | Tissue | qRT-PCR | mean | OS | 2.124 | 1.074 | 4.2 | 0.03 | 20 |
| Giovannetti | 2010 | Italy | R | Pancreatic cancer | 81 | I-IV | Tissue | qRT-PCR | median | OS | 3.1 | 1.2 | 5.3 | 0.003 | 17.3 |
| Faragalla | 2012 | Canada | R | Renal cell carcinoma | 107 | I-III | Tissue | qRT-PCR | 5-fold | OS | 1.75 | 0.87 | 3.51 | 0.11 | 50 |
| Osawa | 2011 | Japan | R | Gastric cancer | 94 | I-IV | tissue | qRT-PCR | median | OS | 1.44 | 0.18 | 14.99 | 0.732 | 37.8 |
| Jung | 2012 | USA | R | Oral cancer | 17 | I-IV | tissue | qRT-PCR | median | OS | 5.31 | 1.39 | 20.38 | 0.015 | 10-174 |
| Karakatsanis | 2013 | Greece | R | Hepatocellular carcinoma | 179 | I-III | tissue | qRT-PCR | mean | OS | 1.716 | 1.419 | 2.075 | 0.01 | 36-144 |
| Ota | 2010 | Japan | R | Breast cancer | 291 | I-III | Bone marrow | qRT-PCR | 2-fold | DFS | 1.04 | 0.71 | 1.48 | 0.853 | 61 |
| Saito | 2011 | Japan | R | NSCLC | 191 | I-II | Tissue | qRT-PCR | median | RFS | 2.66 | 1.47 | 4.83 | 0.001 | 80 |
| Jiang | 2011 | China | R | Melanoma | 86 | I-IV | Tissue | qRT-PCR | median | DFS | 1.008 | 0.818 | 1.378 | 0.086 | 60 |
| Wang | 2012 | China | R | Pancreatic cancer | 177 | III-IV | Serum | qRT-PCR | median | TTP | 1.92 | 1.274 | 2.903 | 0.002 | 20 |
| Caponi | 2013 | Italy UK | R | Pancreatic cancer | 65 | I-III | Tissue | qRT-PCR | median | DFS | 2.3 | 1.16 | 4.56 | 0.02 | 28.4 |
| Amankwah | 2013 | USA | R | Prostate cancer | 65 | I-IV | Tissue | qRT-PCR | median | DFS | 1.99 | 0.7 | 5.64 | 0.2 | 95.1 |
| Faragalla | 2012 | Canada | R | Renal cell carcinoma | 107 | I-III | Tissue | qRT-PCR | 5-fold | DFS | 1.64 | 0.83 | 3.27 | 0.15 | 50 |

a: In study design, R stands for retrospective.
